# Adolescent values for immunisation programs in Australia: A discrete choice experiment

**DOI:** 10.1371/journal.pone.0181073

**Published:** 2017-07-26

**Authors:** Bing Wang, Gang Chen, Julie Ratcliffe, Hossein Haji Ali Afzali, Lynne Giles, Helen Marshall

**Affiliations:** 1 The Robinson Research Institute, University of Adelaide, Adelaide, South Australia, Australia; 2 Adelaide Medical School, University of Adelaide, Adelaide, South Australia, Australia; 3 School of Public Health, University of Adelaide, Adelaide, South Australia, Australia; 4 Vaccinology and Immunology Research Trials Unit (VIRTU), Women's and Children's Hospital, North Adelaide, South Australia, Australia; 5 Centre for Health Economics, Monash Business School, Monash University, Clayton, Victoria, Australia; 6 Institute for Choice, UniSA Business School, University of South Australia, Adelaide, South Australia, Australia; University of Cambridge, UNITED KINGDOM

## Abstract

**Objectives:**

The importance of adolescent engagement in health decisions and public health programs such as immunisation is becoming increasingly recognised. Understanding adolescent preferences and further identifying barriers and facilitators for immunisation acceptance is critical to the success of adolescent immunisation programs. This study applied a discrete choice experiment (DCE) to assess vaccination preferences in adolescents.

**Methods:**

This study was conducted as a cross-sectional, national online survey in Australian adolescents. The DCE survey evaluated adolescent vaccination preferences. Six attributes were assessed including disease severity, target for protection, price, location of vaccination provision, potential side effects and vaccine delivery method. A mixed logit model was used to analyse DCE data.

**Results:**

This survey was conducted between December 2014 and January 2015. Of 800 adolescents aged 15 to 19 years, stronger preferences were observed overall for: vaccination in the case of a life threatening illness (p<0.001), lower price vaccinations (p<0.001), mild but common side effects (p = 0.004), delivery via a skin patch (p<0.001) and being administered by a family practitioner (p<0.001). Participants suggested that they and their families would be willing to pay AU$394.28 (95%CI: AU$348.40 to AU$446.92) more for a vaccine targeting a life threatening illness than a mild-moderate illness, AU$37.94 (95%CI: AU$19.22 to AU$57.39) more for being vaccinated at a family practitioner clinic than a council immunisation clinic, AU$23.01 (95%CI: AU$7.12 to AU$39.24) more for common but mild and resolving side effects compared to rare but serious side effects, and AU$51.80 (95%CI: AU$30.42 to AU$73.70) more for delivery via a skin patch than injection.

**Conclusions:**

Consideration of adolescent preferences may result in improved acceptance of, engagement in and uptake of immunisation programs targeted for this age group.

## Introduction

Adolescence is a time in life that often features risk taking behaviours, however it also provides the greatest opportunity for sustained wellbeing into adulthood. Although adolescents are often treated as younger adults, their views and values are typically overlooked when public health strategies that affect them are being designed.

One of these strategies is immunisation, with adolescents an increasingly important target group for immunisation internationally [1]. Routine immunisation of adolescents provides individual protection and herd protection against vaccine-preventable diseases such as Human Papillomavirus (HPV) and meningococcal disease, boosts the pre-existing but waning immunity (e.g. diphtheria/tetanus/pertussis booster vaccination) and delivers catch-up programs for those who did not receive recommended vaccines during childhood [2]. However, compared to infant and childhood immunisation, the current adolescent immunisation coverage is suboptimal with uptake rates varying between 50% and 80% in high resource countries (e.g. Australia [3] and the United States [4]). Lack of awareness of vaccination recommendations, concerns about vaccine side effects, confusion over immunisation schedules and not actively attending preventive health visits could be barriers to vaccinating adolescents [5, 6]. However, earlier studies in this area have mainly focused on parental or adult preferences for immunisation or on a specific vaccine, such as for HPV, for adolescents [7–13]. Since there is an evidence base to indicate that adolescents are willing to be involved and their attitudes can significantly affect parents' vaccination decisions [5, 14, 15], adolescent immunisation uptake could be improved through better understanding adolescent preferences for vaccination. Understanding their preferences is also important for the development of any vaccination education programs. Such programs can overcome vaccine hesitancy or refusal, and can also provide vaccine providers and health authorities with useful information to inform policy prior to the introduction of any future targeted adolescent vaccine programs.

Discrete choice experiments (DCEs) are commonly used in health economics to elicit participants’ preferences for healthcare programs and policies. The technique uses an attribute based quantitative survey method and draws on elements of random utility theory, consumer theory, and experimental design theory. In DCEs, a number of salient attributes are used to describe characteristics of interventions, and each attribute takes a range of levels. The value (utility) of each scenario is determined by different levels of attributes. Participants trade off risks and benefits among alternative scenarios and express their preferences by choosing their preferred option [16, 17]. Where price is included as an additional attribute, the DCE approach may also be used to estimate individuals’ willingness to pay (WTP) for healthcare interventions [16]. Immunisation acceptance by adolescents may be influenced by a number of factors including severity of illness, side effects, out-of-pocket costs, healthcare facilities where vaccines are administered, mode of administration, vaccine effectiveness and duration of immunity [9, 12]. Adolescents may choose to trade off the potential health benefits against perceived drawbacks of immunisation in the decision making dynamic.

An adolescent-friendly approach, which includes eliciting adolescent views on public health programs that we expect them to engage in, is required if we aim to reduce the barriers to taking part in such programs. Several different methodologies including DCEs have been used previously to assess adolescent values of health states [18, 19]. However, a limited amount of research has been conducted to date to assess adolescent preferences and attitudes towards immunisation program delivery [14, 20–22]. Using an online DCE, this study aimed to investigate adolescent preferences to determine the most important factors influencing their decisions for immunisation.

## Methods

### Survey development

This survey was conducted according to guidelines for the design and conduct of DCE studies in healthcare [17, 23–25].

For this study, it was important to identify a number of relevant and generic attributes that enable participants to make a meaningful judgment regarding adolescent preferences for immunisation. We considered a literature review and expert opinion (interview with a clinician in child and adolescent health and vaccinologist, a health economist/DCE expert, an ethicist and an adolescent health researcher) as the appropriate sources of information. A rapid systematic review was performed by searching titles and abstracts in the PubMed database for DCE studies investigating vaccines preferences. Experts were asked to review the list of attributes derived from the literature review, and the following were identified as appropriate to include in our DCE: disease target [26–29], location of vaccination [9, 13, 30, 31], potential for side effects [8–10, 13, 30–35], vaccine delivery mechanism [10] and price [9, 10, 13, 30, 31, 35, 36]. Since herd protection is an important factor affecting acceptance of vaccines and outcomes of cost-effectiveness evaluations [37], “target for protection” was also added to the attribute list based on the expert opinion. The levels of each attribute were selected as to whether they were plausible and relevant from both the clinical and the policy viewpoint. Based on the range of private vaccine prices in Australia (approximately AU$ 30–200 per dose) and assumption of at least three doses required, price levels of AU$100 and AU$500 were chosen in addition to publicly-funded free vaccination. A previous DCE study found the adolescents’ personal financial situation was significantly associated with their vaccination choice rather than their household financial situation [7]. Considering some adolescents might have already worked full or part-time, we used the term “cost to self (or family)”. Vaccine efficacy was selected as an attribute in a number of previous DCE studies [8–10, 13, 26–34], but not included in our DCE survey as it was not reported as a major contributor to vaccine hesitancy or refusal [38, 39]. Previous research found participants’ decisions to vaccinate were not sensitive to the probability of disease [35]. Therefore neither disease prevalence nor incidence were included in order to reduce participants’ cognitive burden.

A D-efficient (D_z_-error, i.e. zero priors assumed for all variables) design, for main effects only, was developed using Ngene 1.1.2 [40], which yielded 36 choice sets that were further divided into three blocks so as to minimise participants’ cognitive burden. Each participant was randomly assigned to one of the three blocks. One choice question in each block was repeated to check for internal consistency. An example of a choice question is shown in Table 1. Before participants were asked to make a choice between options A or B for each choice question, a detailed explanation of how to choose between alternatives was presented. The possible differences in each hypothetical scenario were listed: 1) disease targeted including mild-moderate illness (unlikely to be fatal), life threatening illness (could be fatal), sexually transmitted infection, or chronic illness; 2) target for protection including the individual (you)–being vaccinated will provide protection against disease affecting adolescents and young adults, or the individual (you) and others–being vaccinated will protect the individual (you) and others by reducing spread of disease to others in the community; 3) price including $0, $100, or $500; 4) setting (location of vaccination) including school/university, GP (i.e. family practitioner), or council immunisation clinic; 5) potential for side effects including rare (1:100,000) but serious (i.e. allergic reaction), or common but mild and resolving (i.e. fever, local redness or swelling); 6) vaccine delivery mechanism including injection (needle), skin patch, or oral dose.

**Table 1:** Example of a DCE question. Please consider that you are making a choice about receiving a vaccine/s for yourself. Of the options in the table below (A or B), please select which option you would choose. Considering the possible scenarios outlined below, which option would you choose?

Features	Option A	Option B
**Disease targeted**	Chronic illness	Mild-moderate illness (unlikely to be fatal)
**Target for protection**	The individual (you)–being vaccinated will provide protection against disease affecting adolescents and young adults	The individual (you) and others–being vaccinated will protect the individual (you) and others by reducing spread of disease to others in the community
**Cost to self (or family)**	$500	$100
**Location of vaccination**	General practitioner (GP)	School/University
**Potential for side effects**	Common but mild and resolving (i.e. fever, local redness or swelling)	Rare (1:100,000) but serious (i.e. allergic reaction)
**Vaccine delivery mechanism**	Oral dose	Skin patch
**Which option would you be more likely to choose?**	**○**	**○**

The questionnaire included a series of socio-demographic questions and 13 DCE choice questions. In addition, two questions in relation to attitudes towards risk in general or with health were measured on an eleven point scale, with zero indicating “not at all prepared to take risk”, and ten indicating “very much prepared to take risk” [41] to assess risk taking behaviours.

The draft survey questionnaire was pre-piloted with a convenience sample of three adolescents and only minor changes were made to ensure adolescents could interpret all questions appropriately. The survey was also pilot tested in 130 participants with approximately 43 participants per block to check feasibility and internal consistency. Seventeen participants (13.1%) failed the internal consistency test. Since the inconsistency rate was comparable to that reported in previous DCE studies [42, 43], no revisions were made to the DCE survey.

### Sample size and study population

Calculation of optimal sample sizes is complex as it depends on the true values of the unknown parameters estimated in the DCE models [17]. However, as a rule of thumb suggested by Orme [44], a sample size of 300 would be desirable for a main effects model based on the number of choice sets, alternatives and analysis cells. We aimed to recruit 20 participants per choice set resulting in 720 adolescents aged between 15–19 years, which would provide more statistical power with a sample size larger than in similar adolescent DCE studies described in the literature to date [7, 8, 32].

Potential participants were identified via Pureprofile (https://www.pureprofile.com/au/), an online market research company. Pureprofile was contracted to host and distribute the survey invitation to parents on their database who had children aged between 15–19 years and resided within Australia. Interested parents were provided with an electronic information sheet describing the study. Parents were then asked whether they had an adolescent who would be willing to complete the survey. Subsequent to parent and adolescent dyad consent to participate in the study, adolescents were then guided through the online survey by screen prompts. In recognition of the time spent completing the DCE survey, account holders of adolescents who participated received a small financial reward (AU$3.25).

### Statistical analysis

The Socio-Economic Indexes for Areas, Index of Relative Socioeconomic Disadvantage 2011 (SEIFA IRSD) [45] was used to categorise socio-economic status as into tertiles: low (1st–33rd percentile), medium (34th–66th percentile) and high (67th–100th percentile). SEIFA ranks residential areas in Australia according to relative socio-economic disadvantage based on information from the five-yearly Census. Student’s t-tests and χ^2^ tests were used to compare means and proportions between two subgroups, respectively. Participants who failed the internal consistency test were excluded from the analysis and a sensitivity analysis was conducted by including participants who failed the test.

DCE data were analysed using a mixed-logit model which accounts for preference heterogeneity. The price attribute was treated as a continuous variable and dummy-variable coding was used for all other attributes. The model fit to the utility function was:

$$\begin{array}{ll} U_{itj}= & \left(\beta \text{1}+\eta_{1i}\right)\text{ life threatening illness}+\left(\beta \text{2}+\eta_{2i}\right)\text{ sexually transmitted infection}\\ & +\left(\beta \text{3}+\eta_{3i}\right)\text{ chronic illness}+\left(\beta \text{4}+\eta_{4i}\right)\text{ protect you\&others}\\ & +\left(\beta \text{5}+\eta_{5i}\right)\text{ school/university}+\left(\beta \text{6}+\eta_{6i}\right)\text{ GP}+\left(\beta \text{7}+\eta_{7i}\right)\text{ common side effects}\\ & +\left(\beta \text{8}+\eta_{8i}\right)\text{ skin patch}+\left(\beta \text{9}+\eta_{9i}\right)\text{ oral dose +}\left(\beta \text{10}+\eta_{10i}\right)\text{ price}+\;\varepsilon_{itj} \end{array}$$

*U*_*itj*_ describes the utility of a hypothetical vaccine scenario, *i* derives from an individual choosing alternative *j* in choice question *t*, *β*_*i*_ is a vector of coefficients reflecting participants’ preference for each attribute level on average, *η*_*i*_ indicates the individual’s specific preference (i.e. a random effect), and *ε*_*itj*_ is a random error term describing the unmeasured variation in participants’ preferences. We assumed coefficients of all attribute levels were independent and randomly distributed with a Normal distribution. A positive (negative) and significant coefficient indicates a positive (negative) preference for a specific attribute level. The coefficient estimates (or preference weights) can also be used to compare relative importance between different levels of the same attribute or between levels of completely different attributes [11].

WTP represents a monetary measure of participants’ valuation for a change in the level of the attribute of interest. It is the ratio of the coefficient for a certain attribute and the price coefficient (- $\frac{\beta_{k}}{\beta_{c}}$ where *β*_*c*_is the price coefficient and *β*_*k*_is the coefficient for attribute *k*). The positive and negative results indicate theoretically to what extent the participants and their families would be willing to pay/to be compensated for an attribute level. The 95% confidence intervals were estimated using the Krinsky Robb (parametric bootstrap) method [46]. WTP estimates do not represent market prices participants and their families wanted to pay for the various attributes of a hypothetical vaccine. All statistical analyses were performed in Stata version 14.1 [47].

### Ethics

This study was approved by the Women’s and Children’s Health Network Human Research Ethics Committee in Adelaide, Australia. This study has not been registered in a clinical trial registry because it was not a clinical trial and therefore registration was not required.

## Results

A total of 800 adolescents (age range 15–19 years) were enrolled and completed the survey between December 2014 and January 2015 (S1 Dataset). Females were slightly predominant (54.9%) in the study population. Of the participants, 90.0% were born in Australia, with approximately 97.8% non-indigenous (Table 2). Enrolment was initially planned to be stratified by state and gender. Due to difficulties in recruiting adolescent participants in smaller states or territories such as the Northern Territory (NT) and Australian Capital Territory (ACT), enrolment did not strictly adhere to the original regional quotas. Except for NT and ACT, participants were reasonably representative of the adolescent population of each state.

**Table 2:** Demographic characteristics of the study population.

	All (N = 800)		Participants who passed the consistency test only (N = 695)		Participants who failed the consistency test only (N = 105)		P value
	Mean	SD	Mean	SD	Mean	SD	
Age (years)	17.10	1.42	17.11	1.42	17.08	1.39	0.839
Household Size (people)	4.09	1.41	4.05	1.35	4.30	1.77	0.088
Risk attitudes							
In general	5.20	2.32	5.12	2.26	5.71	2.64	0.015
For health	4.11	2.61	4.01	2.53	4.79	3.00	0.004
	**N**	**%**	**N**	**%**	**N**	**%**	
Gender							
Male	361	45.13	317	45.61	44	41.90	0.477
Female	439	54.88	378	54.39	61	58.10	
Completed High School	445	55.63	391	56.26	54	51.43	0.353
Born in Australia	720	90.00	629	90.50	91	86.67	0.222
Aboriginal or Torres Strait Islander	17	2.13	16	2.30	1	0.95	0.371
Socio-economic Status							
Low (1st–33rd percentile)	203	25.50	183	26.48	20	19.05	0.008
Medium (34th–66th percentile)	252	31.66	205	29.67	47	44.76	
High (67th–100th percentile)	341	42.84	303	43.85	38	36.19	
State							
NSW	257	32.13	224	32.23	33	31.43	0.224
VIC	201	25.13	173	24.89	28	26.67	
QLD	166	20.75	149	21.44	17	16.19	
SA	70	8.75	55	7.91	15	14.29	
WA	79	9.88	68	9.78	11	10.48	
TAS	16	2.00	16	2.30	0	0.00	
ACT & NT	11	1.38	10	1.44	1	0.95	

### DCE results

Participants who failed the consistency test were excluded from the analysis (N = 105, 13.1%), generating a useable total sample of 695 adolescents (86.9%) for main DCE analysis. Except for socio-economic status (SES) and risk taking attitudes, there were no significant differences between the participants who passed versus those who failed the consistency test. Those who were excluded were more likely to reside in an area with medium SES (p = 0.008) and exhibited higher general (p = 0.015) and health risk attitudes (p = 0.004).

**Fig 1:**
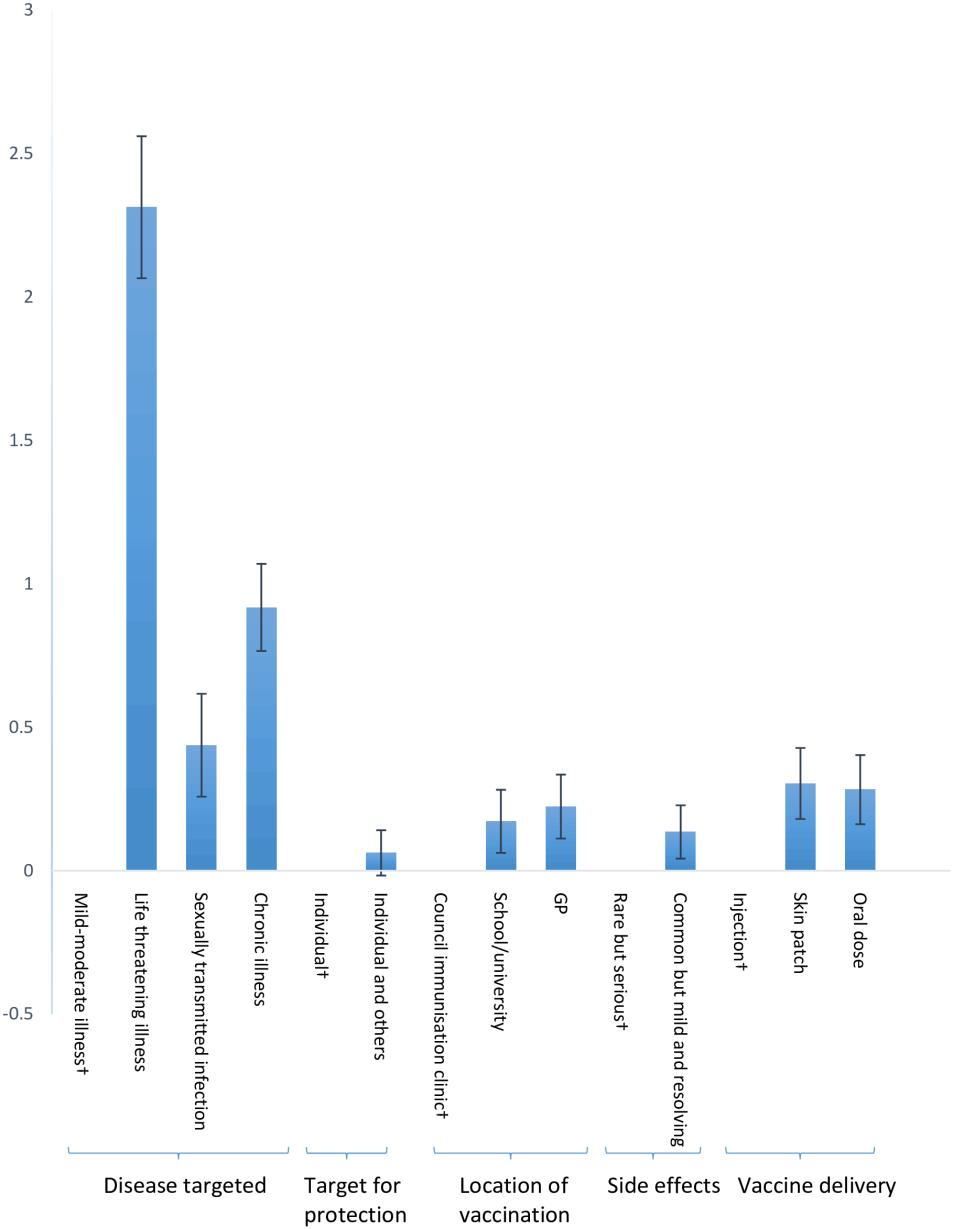
Preference weights for nonmonetary attributes. † Reference (omitted) level for each attribute.

The vaccination in the case of a life threatening illness (p<0.001) had the highest preference weight when comparing with a mild-moderate illness (Fig 1 and Table 3). Changing vaccination targeting from a mild-moderate illness to a life threatening illness could yield 17 times (2.314 ÷ 0.135) as much as utility as changing from “rare but serious” to “common but mild and resolving” side effects. Other stronger preferences were observed for vaccination treating a chronic illness (p<0.001) and a sexually transmitted infection (p<0.001) with common but mild and resolving side effects (p = 0.004) and delivery via a skin patch or oral dose (p<0.001) compared with their reference levels. Despite the success of adolescent school-based vaccination, participants were more willing to be vaccinated by GPs (p<0.001). Lower price vaccinations were also preferred (p<0.001).

**Table 3:** Mixed logit estimates on vaccination preferences.

Attributes	Coefficient	SE	P value	SD	SE	P value
**Disease targeted**						
Mild-moderate illness ^a^						
Life threatening illness	2.314	0.126	<0.001	1.909	0.135	<0.001
Sexually transmitted infection	0.437	0.092	<0.001	1.408	0.101	<0.001
Chronic illness	0.918	0.078	<0.001	0.418	0.178	0.019
**Target for protection**						
Individual ^a^						
Individual and others	0.062	0.040	0.126	0.113	0.103	0.274
**Location of vaccination**						
Council immunisation clinic ^a^						
School/university	0.172	0.056	0.002	0.267	0.125	0.033
GP	0.223	0.057	<0.001	0.290	0.120	0.016
**Potential for side effects**						
Rare but serious ^a^						
Common but mild and resolving	0.135	0.047	0.004	0.558	0.067	<0.001
**Vaccine delivery mechanism**						
Injection ^a^						
Skin patch	0.304	0.063	<0.001	0.566	0.091	<0.001
Oral dose	0.283	0.062	<0.001	0.302	0.127	0.018
**Price**	-0.006	<0.001	<0.001	0.006	<0.001	<0.001
Log likelihood	-3893.719					
Number of participants ^b^	695					
Number of observations ^c^	16680					

Notes: SE—standard errors. SD—standard deviation. For all random coefficients, normal distribution was used. Price attribute was included as a continuous variable; all other attributes were dummy coded.

^a^ Reference (omitted) level for each attribute

^b^ A total of 800 adolescents completed the survey. Participants who failed the consistency test (N = 105) were excluded from the main analysis reported in this table.

^c^ In total, 16680 scenarios (2*12*695) were assessed, with 12 choice sets per participant and each consisting of a choice between two alternative vaccination programs (A and B).

With the exception of one coefficient (for vaccination protecting you and others (p = 0.274)), the standard deviations (SDs) of other random coefficients were statistically significant, which indicated preference heterogeneity was present for those attribute levels.

A sensitivity analysis was performed by including participants who failed the consistency test and no significant impact was observed. Subgroup analyses were conducted with regard to SES, risk taking attitudes and participants’ intention to be vaccinated, and the results were broadly consistent between subgroups.

### Willingness to pay

Participants suggested that they and their families would be willing to pay AU$394.28 (95%CI: AU$348.40 to AU$446.92) more for a vaccine targeting a life threatening illness than a mild-moderate illness, AU$37.94 (95%CI: AU$19.22 to AU$57.39) more for being vaccinated at a family practitioner clinic than a council immunisation clinic, AU$23.01 (95%CI: AU$7.12 to AU$39.24) more for common but mild and resolving side effects than rare but serious side effects, and AU$51.80 (95%CI: AU$30.42 to AU$73.70) more for delivery via a skin patch than injection (Table 4).

**Table 4:** Willingness to pay (AU$) for vaccination (based on mixed logit estimates).

Attributes	Willingness to pay (AU$)	95%CI
**Disease targeted**		
Mild-moderate illness ^a^		
Life threatening illness	394.28	348.40, 446.92
Sexually transmitted infection	74.43	44.10, 106.37
Chronic illness	156.35	129.76, 185.55
**Target for protection**		
Individual ^a^		
Individual and others	10.53	-3.29, 24.52
**Location of vaccination**		
Council immunisation clinic ^a^		
School/university	29.33	10.70, 48.54
GP	37.94	19.22, 57.39
**Potential for side effects**		
Rare but serious ^a^		
Common but mild and resolving	23.01	7.12, 39.24
**Vaccine delivery mechanism**		
Injection ^a^		
Skin patch	51.80	30.42, 73.70
Oral dose	48.25	27.95, 69.82
**Number of observations**	16680	

Notes: Confidence interval (CI) was calculated based on the Krinsky and Robb bootstrap method (with 10,000 replications). Price attribute was included as a continuous variable; all other attributes were dummy coded.

^a^ Reference (omitted) level for each attribute.

## Discussion

This DCE has identified preferences of Australian adolescents for immunisations providing protection against a life threatening illness, causing common but mild and resolving side effects, being administered by a medical practitioner and delivered via a skin patch at a lower price. To our knowledge this is the first study to investigate adolescent preferences for immunisation delivery using a DCE design. Because comparable data are lacking, we have reviewed literature for DCE studies associated with a specific vaccine in both parental and adolescent populations.

Fatal diseases were the most vital decisive factor in adolescent vaccine acceptance. Another DCE study reported people valued prevention targeting a serious illness higher than cure [48]. This suggests that vaccines targeted towards a fatal illness could achieve high and sustainable vaccine coverage, for example, adolescent vaccines for meningococcal disease. Given the National HPV Vaccination Program started almost ten years ago [49], somewhat surprisingly, our study participants were not strongly in favour of STI vaccines which may indicate lack of awareness of HPV being a STI. Parental studies reported similar results that a sexual mode of transmission had minimal impact on STI vaccine acceptability [27, 28]. Moreover, only 13% of adolescent girls were concerned about HPV in an HPV study conducted in the United States [7]. Perceived transmission risks or severity of STI might be quite low in adolescents, which resulted in a relatively lower estimated coefficient on STI compared to life threatening and chronic illnesses. Although previous research indicated participants’ choices to vaccinate were not sensitive to the probability of disease [35], assumptions made by participants about the incidence of the disease prevented might influence their preferences. The results of disease severity may be interpreted with caution, for example, we cannot definitively conclude that adolescents indicated they and their families would be willing to pay AU$394 more for a vaccine against a life-threatening but potentially very rare disease, as compared with a mild-moderate, but common one. Further research may be warranted to tease out the effects of the disease incidence versus disease severity.

Adolescent immunisation preferences were also influenced by the severity of potential side effects. Previous research only assessed impact of the frequency of severe reactions [9, 30, 33, 34]. Our study compared preferences between two common occurrences of side effects: rare but serious versus common but mild. Compared to the frequency, the severity of side effects may play a more important role in the decision making process.

Although participants still showed positive preferences for the school or university, GP clinics were their stronger location preference in our study. In Australia, adolescent school-based vaccination has demonstrated advantages over community or private sectors and achieved a higher coverage rate [1]. However, a lack of awareness or miscommunication might affect their preference for school-based vaccination. More chances to communicate with GPs and understanding benefits and risks of the vaccination might explain why participants preferred GP clinics in our study. Similar to our finding, a varicella vaccination study reported that the provision of vaccination at schools did not affect parents' choices whether or not to immunise their child [31].

Our study also found that price was an important attribute driving preferences which is in line with previous research [9, 11, 31, 50, 51]. Recommended but non-publicly funded vaccines were more likely to be refused by parents due to the price [50]. If a vaccine was not included on the National Immunisation Program Schedule, vaccine prices would definitely be a financial barrier to successful implementation of an immunisation program. Although adolescents usually would not have any income or direct out-of-pocket costs for their medical care, a DCE study assessing WTP for a meningococcal B vaccine in Australia found a consistent pattern of results at all attributes and levels between adolescents and adults [12]. That financial barrier would still affect adolescent actual decision making when it comes to receipt of vaccines.

The strength of this study is identification of adolescent preferences for immunisation programs using a DCE survey, which allows us to investigate multiple factors influencing vaccination decision and trade-off between attribute levels. Based on a large national sample of adolescents, our study produced meaningful and robust estimates. There were some limitations to our study. Prior qualitative work was not conducted to select attributes and levels. It is possible that some potentially important attributes were omitted from the design of this study (e.g. disease incidence). Since preferences were measured to establish which components define the most preferred vaccine program from an adolescent perspective, an opt-out option was not provided and participants were forced to choose between two alternatives. Whilst it may be argued that including an opt-out option might reflect the decisions of participants in real-life settings, the opt-out option might be selected by participants to avoid making difficult trade-offs on attribute levels, thereby decreasing the precision of parameter estimates [52]. However, the inclusion of an opt-out option may provide more information about trade-offs between vaccination and no vaccination. Furthermore the opt-out option would have enabled the prediction of probabilities of take-up of different vaccine scenarios [17, 31] and might correct the WTP value for the probability of people opting out [53]. Further research is required to explore the implications of including an opt-out option in this context. Their identification and vaccination status cannot be verified, which may affect internal validity of the study. As our participants were adolescents who might not be financially independent, the WTP values in our study are a mix of personal values and perception of what their family would sacrifice and therefore WTP may not be interpreted in the conventional way. Finally, since this is a survey-research study and only participants with internet access could be enrolled, the sample may not be entirely representative of the general population of adolescents due to a higher percentage of adolescents from areas of high/medium SES with higher educational levels.

Understanding barriers and facilitators to immunisation is an important step to improve the uptake of adolescent immunisation. Our study showed adolescents’ vaccine decisions were driven by disease types, healthcare facilities where vaccines were administered, severity of side effects and vaccine delivery methods. The study results can provide useful information on adolescent views, values and preferences for vaccination to health authorities, vaccine providers, immunisation educators and healthcare providers. Strategies to increase immunisation uptake among adolescents may include providing adolescent-tailored education programs, lowering out-of-pocket costs, and offering vaccinations outside of schools in “complementary” settings (e.g. GP clinics). This study evaluating adolescent preferences for immunisation may be used to inform any future health economic studies for individual vaccines before they are publicly available. For example, the predicted high rates of vaccination against fatal illnesses, may positively affect outcomes of health economic evaluation. Our study results may also be used to develop adolescent specific immunisation education programs. When designing an education program for adolescent immunisation, these factors, particularly the relative severity of the disease, should be clearly explained to adolescents.

## Supporting information

S1 Dataset DCE data from participants in this study. (XLSX)

## Abbreviations

ACT: Australian Capital Territory
DCE: Discrete Choice Experiment
GP: General Practitioner
HPV: Human Papillomavirus–
NT: Northern Territory
SD: Standard Deviation
SEIFA IRSD: Socio-Economic Indexes for Areas,Index of Relative Socioeconomic Disadvantage
SES: Socio-Economic Status
STI: Sexually Transmitted Infection
